# Early detection and intervention of psychosis in children and adolescents in Zurich, Switzerland: Clinical Data from 2017-2022

**DOI:** 10.1192/j.eurpsy.2023.2284

**Published:** 2023-07-19

**Authors:** M. Franscini, N. Traber-Walker, F. Probst, M. Gerstenberg, S. Walitza

**Affiliations:** 1 Department of Child and Adolescent Psychiatry and Psychotherapy, University of Zurich, Zurich, Switzerland

## Abstract

**Introduction:**

The construct of a clinical high-risk (CHR) state of psychosis has been established to describe potentially prodromal symptoms which typically appear during adolescence and young adulthood. This is a very sensitive developmental period and the clinical high risk (CHR) is associated with increased functional impairment. To address the specialities in the care for this patient population a specialized outpatient care unit for early intervention in psychosis at the Department of Child and Adolescent Psychiatry and Psychotherapy, Psychiatric University Hospital, of the University Zürich (CAPS) is established. The interdisciplinary team (psychiatrists and psychologists) supports children and adolescents with psychotic disorders or at clinical high risk for developing psychosis. The early intervention service offers specialized assessment, treatment and case management for minors with a first psychosis or CHR-state in an outpatient or inpatient setting as well as by day clinic care.

**Objectives:**

The evaluation main objective was to get a better understanding about this vulnerable patient group. Therefore we analysed the clinical data about CHR-state, comorbid diagnosis, treatment, medication and hospitalisation of the patients who entered the service for early intervention in psychosis.

**Methods:**

Participants who entered the service for early intervention in psychosis were followed up in the years 2017-2021 and descriptive analysis was used to summarize the data. For the evaluation of the risk construct the participants have been classified in “no increased risk”, “CHR” or “early onset psychosis” (EOP). Additionally, ICD diagnosis, demographics and treatment (medication, psychotherapy, treatment setting) were assessed. Therapy was either psychotherapy and/or group training called DBT2P (Dialectical behavioral group training for adolescents, to prevent psychiatric disorders). Additionally, the use of a smartphone application “Robin Z”(add-on treatment tool to support the patients between the sessions) was assessed.

**Results:**

In the last five years we saw 300 patients (112 female, mean age 15.7) who sought the care unit for early intervention. The evaluation of the risk showed that 44 patients had no increased risk, 205 were classified with a CHR and 51 fulfilled the criteria of an early onset psychosis (18.5%). Most of the patients showed comorbid diagnosis, mainly depressive disorders (42%). The data about the treatment will be analyzed for the congress.

**Conclusions:**

Despite clinical implications, there is little data about early detection and early intervention in psychosis for children and adolescent. Therefore, the evaluation of the clinical data of the CAPS is of clinical importance and expected to add essential information in the fields of prevention and early intervention in psychosis.

**Disclosure of Interest:**

None Declared

